# Scalable multimodal approach for face generation and super-resolution using a conditional diffusion model

**DOI:** 10.1038/s41598-024-76407-9

**Published:** 2024-11-08

**Authors:** Ahmed Abotaleb, Mohamed W. Fakhr, Mohamed Zaki

**Affiliations:** 1 Computer Engineering Department, Arab Academy for Science Technology and Maritime Transport, Cairo, 2033 Egypt; 2 College of Engineering, Al-Azhar University, Cairo, 11884 Egypt

**Keywords:** Scalable multimodal approach, Speech conditioned face generation, Speech conditioned face super-resolution, Diffusion probabilistic models, Speaker embeddings, Computer science, Information technology

## Abstract

Multimodal Conditioned face image generation and face super-resolution are significant areas of research. To achieve optimal results, this paper utilizes diffusion models as the primary engine for these tasks. This paper presents two main contributions: (1) “Speaking the Language of Faces” (SLF): a flexible, modular, fusion-less and architecturally simple multimodal system. (2) A Scalability scheme and a sensitivity analysis which can assist practitioners in system parameter estimation and feature selection. SLF consists of two main components: a feature vector generator (encoder), and an image generator (decoder) utilizing a conditional diffusion model. SLF can accept various inputs, including low-resolution images, speech signals, person attributes (age, gender, ethnicity), or any combination of these. Moreover, Scalability based on conditional scale values is utilized. The implementation of SLF has confirmed its versatility (e.g., speech to face image generation, conditioned face super-resolution). We trained multiple system versions to conduct a sensitivity analysis and to determine the influence of each individual feature on the output image. Consequently, speaker embeddings have proven to be sufficient audio features for our task. It was also found that the effects of audio signals are profound and are more pronounced than those of the low resolution images (8 × 8), whose effects are still significant. The effect of gender, ethnicity and age were found to be moderate. On another note, conditional scale values significantly impact the system’s behavior and performance.

## Introduction

For a long time, the task of person recognition, which is a significant problem in security, military and cognitive applications, has been tackled as a uni-modal (image only or voice only) problem. The current process of speaker recognition involves querying a person’s voice features against a database of voice features associated with known identities^1^. Likewise for face recognition^2^. However, humans use both auditory and visual cues to recognize other humans, and they are able match voices to faces and faces to voices.

**Fig. 1:**
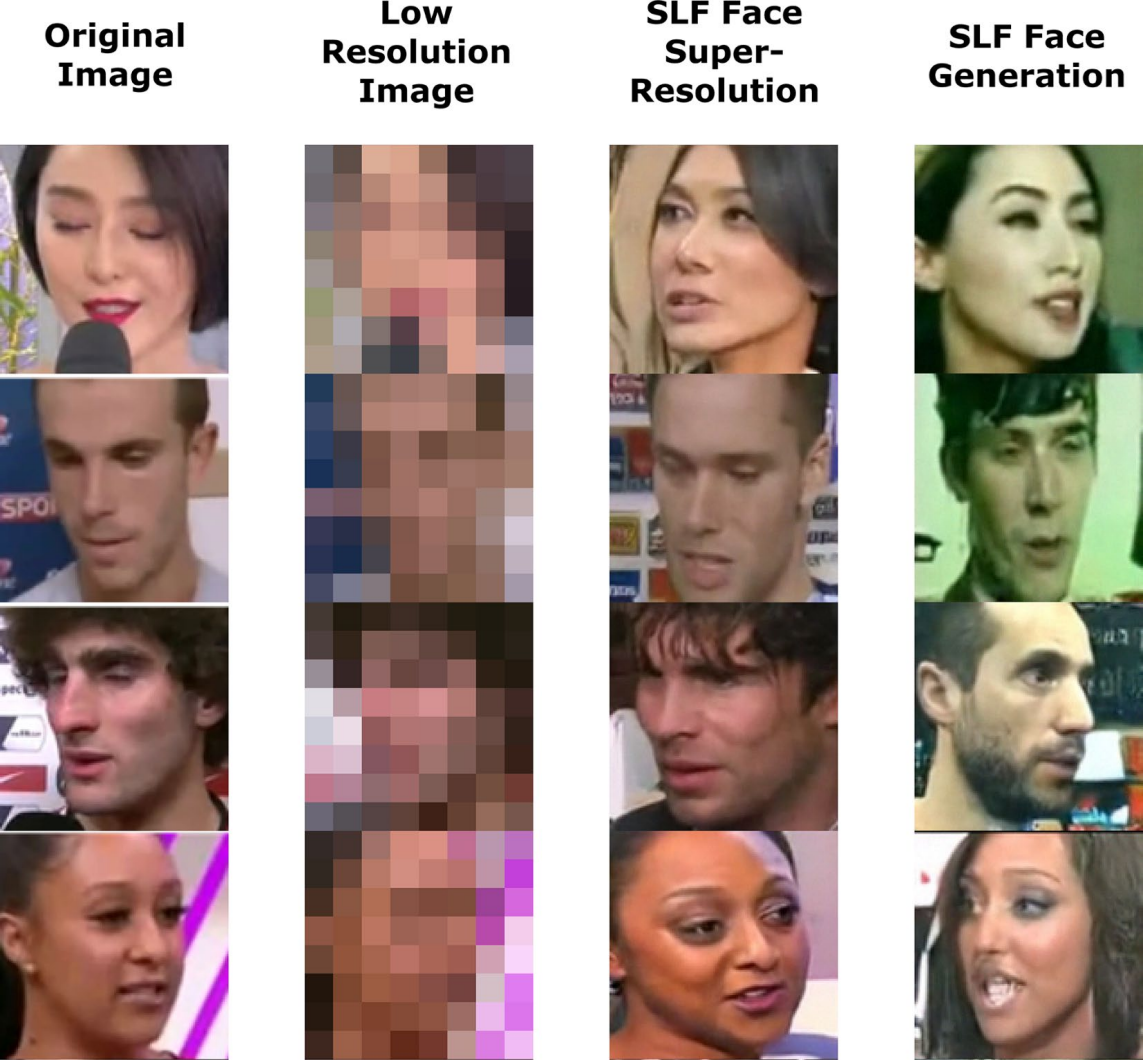
A sample of the output images of a speech conditioned SLF face super-resolution system and a speech conditioned SLF face generation system.

Recently, several researchers have studied using a combination of low resolution images and speech signals to achieve better results in person recognition^3–11^. This is done by either generating face images from speech signals or performing super-resolution on blurry face images and then performing face recognition. Most employed generative adversarial networks, but recently diffusion models^12–16^ have been used for this purpose as well^10,11^. However, diffusion model performance differ by model size, which is indicated by the number of parameters. Scalability studies of diffusion models reveal that small models are preferable in some case studies, while larger models are preferred in others^15,16^. In all cases, the model size is measured by its numbers of parameters, which is not always suitable.

This paper aims at building such systems, which we refer to as “Speaking the Language of Faces (SLF)” using de-noising diffusion models^12–14^, which have recently seen great success in text to image tasks^17–19^. SLF systems incorporate features in a straightforward fusion-less way, and are trained to use any one combination of different input features extracted from speech and face image data, as well as others, to generate their output. This paper also aims to investigate the effects of various audio features, visual invariants and other characteristics on the quality and performance of such multimodal systems, so that the designers of these systems can focus their attention on the factors that affect the system in a beneficial way. A sample of SLF output is shown in Fig. 1.

In this paper, we also study a new measure for scalability, which is the conditional scale value. This value specifies how strongly the input values influence the generation process in a diffusion model. Our model is trained once without specifying the conditional scale value. However, in the operational phase, we specify a specific value. Accordingly, the model performance differs depending on the conditional value chosen. This is taken as a better scalability measure than model size, which requires training different models with different sizes.

Most Speech conditioned face generation and face super-resolution system have employed the traditional models of image generation namely auto-encoder models and GANs, and devise a non-standard way to input speech to the image generation model^3–5^, while in this paper we employ a de-noising probabilistic diffusion model. The model input includes standard features extracted from speech such as speaker embeddings, classical audio features, language spoken, as well as, the low-resolution image of the person (in the case of super-resolution) that we define as an image guide. Additionally, optional attributes such as gender, ethnicity and age can also be included.

**Fig. 2:**
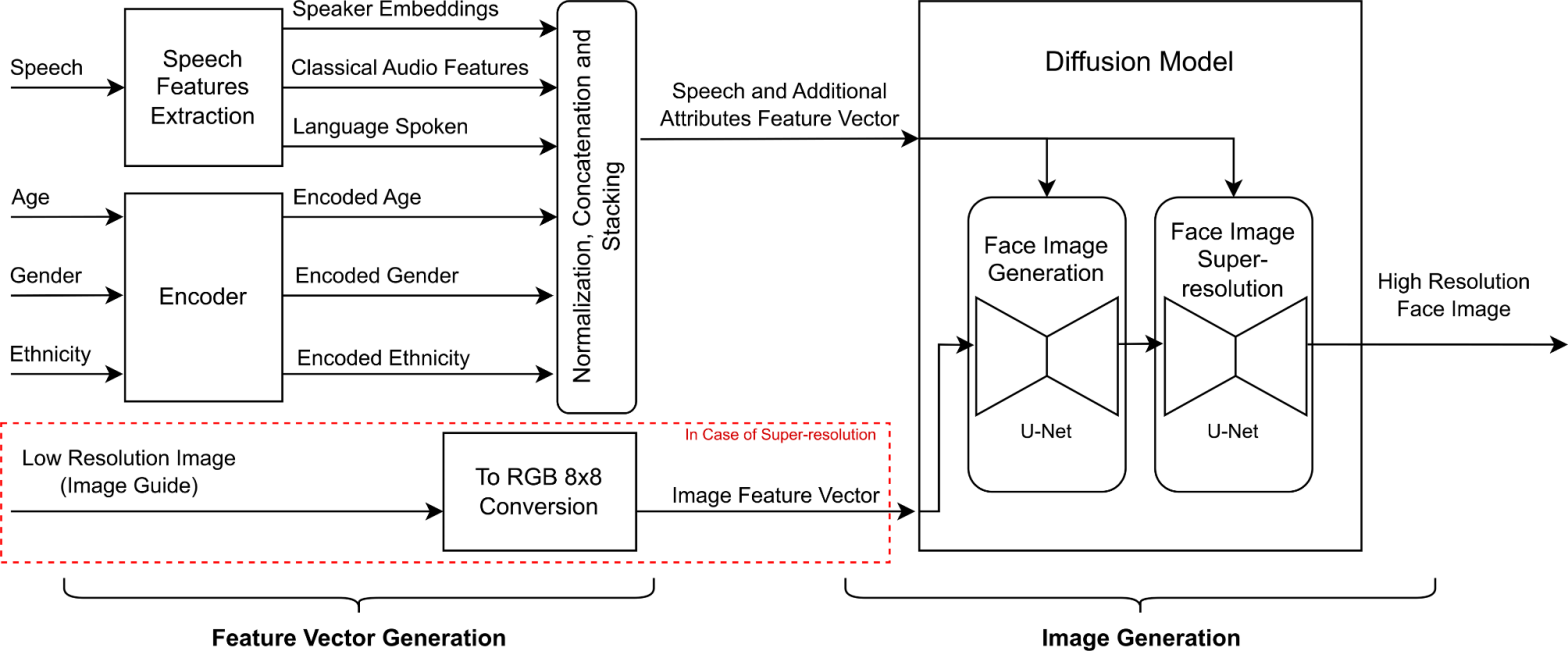
An overview of our SLF system architecture. Age, gender and ethnicity are optional inputs.

SLF consists of two main components: (1) a feature vector generation component and (2) an image generation component. In the first component, a feature vector is generated, while in the second component, a diffusion model is utilized to achieve image generation. SLF system architecture is shown in Fig. 2.

The contributions of this paper can be summarized as follows:

- Building the SLF system as both a multi-modal face generation and face super-resolution system that can accept a combination of various features, which might include low resolution images, speech features and other optional additional features. These features are incorporated in a flexible, modular and fusion-less way, bypassing the complexity of fusion networks. This allows for easy integration of other modalities and replacement of feature extraction models. In the case of speech data, we have opted to use standard audio features.
- Additional model scalability capability, that can enhance the system usability and practicability based on extensive experimental work, is presented, since altering the conditional scale values greatly affects the system behavior and performance. Conditional scale value based scalability is used instead of traditional parameter amount based scalability because of its greater usability and lower cost.
- We trained various versions of the SLF system to complete a thorough ablation study to quantitatively determine the influence of each individual feature set on the output image. It is shown that the effects of the audio signals are profound and are more pronounced than those of the low resolution images (8 × 8), whose effects are still significant, and that the speaker embeddings can be relied upon as sufficient audio features for our task. Moreover, the effects of gender, ethnicity and age on the output image are moderate.

This paper is organized as follows: “Related work” demonstrates the related work in the relevant areas. “The proposed system” details the proposed method, the system architecture and the training stages. In “Results and discussion”, the results are presented and discussed, and a thorough ablation study is presented. The relative position of our system performance against other systems in the literature is also highlighted, as well as, its limitations and ethical considerations. Finally, in “Conclusion”, the conclusions are stated.

## Related work

### Speech to face generation

The generation of face images based on the user’s speech has been addressed in several research works, most important of which is the work done by Oh et al.^3^ which, according to the authors, was the first research work to devise a model for generating faces from speech. The authors employed a speech encoder and a face decoder for that end. The speech encoder attempts to generate VGG facial embeddings, while the face decoder attempts to generate faces from these facial embeddings.

Duarte et al. employed a generative adversarial network instead of a face decoder to generate the face image^5^. Duarte el al. also manually curated a dataset called the “Youtubers” dataset specifically to train this model, and which was publically released. Wen et al. solved this problem as a cross-modal task (speech, face image)^9^. A voice embedding network was used to encode the speech into a voice embedding to be used as an input to a GAN model.

Rather than use speech to generate a face image, Meishvili et al. created a system to perform extreme super-resolution on an 8 × 8 low resolution face image to generate a 128 × 128 high resolution face image utilizing speech data of the speaker^7^. This was done by first generating the latent representation of both the audio signal and face image, then fusing them, and finally generating the high resolution face image via a GAN model. Sun et al. actualized a system that not only generated the face of a speaker through speech but also animates it as well^6^. The authors used techniques such as contrastive learning and curriculum learning (increasing difficulty) for that end.

Focusing on the speech analysis part of the model, BAI et al. introduced an embedding fuser in between the speech encoder and the face decoder^4^. The rationale behind this was to erase the influence of speaker-independent features from the output of the face decoder such as the meaning of speech and emotion.

With regards to handling more inputs to the system apart from speech and low resolution images, Abdeen et al. used a CGAN model to generate the face image conditioned on both speech, and age and gender characteristics^8^.

Turning away from GAN models for image generation, Kato & Hashimoto introduced a novel system for speech to face image generation compromised of a CNN based speech encoder, DDPM based face decoder and super-resolution model^10^. Likewise, Wang et al. proposed a speech-to-face generation framework, that utilizes a speech-conditioned latent diffusion model, called SCLDM^11^. Wang et al. also employed collaborative pre-training with contractive learning for the speech encoder and the facial encoder; this means that the model learns to understand that a certain age or gender in speech should correspond to a certain appearance in a face.

It is apparent that de-noising diffusion probabilistic models, standard speaker derived features namely speaker embeddings and standard classical audio features haven’t seen enough use in speech conditioned face generation and speech conditioned face super-resolution systems. High flexibility systems that can work upon different combinations of input in a fusion-less and architecturally sound similar way for both face generation and face super-resolution are not pronounced enough in the literature. It is also observed that ablation studies focused on the influence of various inputs on the output images are relatively absent, as are diffusion-centric scalable systems.

### Multimodal conditioned face image generation

Multimodality integrating text, images, masks, semantic labels or sketches to synthesize face images have been demonstrated in the literature.

Xia et al. proposed a framework for text guided face image generation and manipulation using a GAN model^20^. This framework is able to generate high resolution face images from a set of multimodal inputs (images, text, sketches and semantic labels). It relies on manipulating the latent variables of a StyleGAN model^21^ to generate a new or altered face image.

Nair et al. have formulated an approach for multimodal image generation, including face image generation, using multiple standalone diffusion models each trained for a certain sub-task and associated with a certain modality^22^. During sampling, their corresponding outputs are combined. This approach accepted text, semantic labels and sketches.

Ham et al. have devised a method to take advantage of already trained image generation diffusion models and adding the capability for multimodal synthesis to them^23^. This is done by modulating the sampling process through multimodal conditioning modules (MCM), so as to insert information from other inputs into the output image. This method accepted segmentation maps, sketches and texts as input. A similar method was proposed by Huang et al.^24^, but instead of multimodal conditioning modules, a dynamic diffuser was proposed, which selectively enhances or suppresses the influence of a given modality. Huang et al.’s work utilized pre-trained diffusion models and was shown to work with images, texts and masks.

Ren et al. have presented a flexible, scalable and adaptive face image synthesis model conditioned on a combination of text descriptions, masks, poses, expressions, lightning and sketches^25^. This model was built upon a diffusion model, and by using uni-modal training with modal surrogates and adapting its processes based on entropy, it ensures high-quality outputs and robust inter-modal collaboration.

It is evident that there an absence of incorporating audio data in current multimodal face image generation techniques, where voice helps synthesize the face image of the speaker. Flexible solutions that can work with any modality, which are architecturally simple and which are scalable are too few in the literature. This work aims to solve these problems.

### Biometric image generation

Image generation and reconstruction techniques for various non-facial biometric body parts such as palms, irises and fingers have been demonstrated in the literature for goals that include inversion or reconstruction attacks against various recognition systems^26^. These attacks aim to reconstruct the required biometric image from obtained or intercepted templates. These templates are the features by which the matching process in various recognition systems are conducted.

Yan, Wang et al. have devised an approach for reconstructing palm images from intercepted deep or handcrafted templates based on a modified progressive GAN (ProGAN) trained using a Scale-Adaptive Multi-Texture Complementarity (SAMTC) loss^27^. A different approach is utilized by Yan, Leng et al. in which the region of interest (ROI) of the palmprint matching algorithm is embedded into a carrier hand image using a pretrained Contrastive Arbitrary Style Transfer network along with a pretrained inpainting technique^28^.

While inversion and reconstruction attacks aim to reverse the feature extraction process that pertains to a single modality, our goal is to convert one modality into another (Face Generation), or to enhance one modality using different ones (Face Super-Resolution). This signifies the multi-modality aspect of our work.

### De-noising diffusion probabilistic models

Diffusion models are a class of image generation models^12^ that are currently being extensively used in unconditional and conditional image synthesis. It was shown that they are able to generate images of higher quality than those created by the then state of the art models (GANs^13,14^). Of substantial importance to our work is the current prevalent use of diffusion models in text to image and in-painting tasks^17–19,29^, which—in several ways—is comparable to speech to face image tasks.

The most important of these works and that which inspired our proposed model is the work done by Saharia et al. which cultivated in a text to image system by the name of “Imagen”^17^, in which cascaded DDPM conditioned on text embeddings from a large pre-trained text encoder were developed.

### Speaker embeddings

Speaker embeddings are feature vectors which encapsulate a speaker’s unique characteristics representing a certain speaker and differentiating him/her from other speakers^30,31^. Speaker embeddings are extracted from a speaker’s speech data and are used in tasks such as speaker verification, speaker recognition and speaker diarization. Speaker embeddings’ differentiating power in speaker recognition tasks are speculated to be useful in our research work.

## The proposed system

In this section, the SLF system is elaborated in detail. In “System overview” the system overview is presented. In “Feature vector extraction” the feature vector extraction part of the system is discussed, while in “Image generation” the image generation part is considered. In “SLF algorithm”, the SLF Algorithm is formulated. We compare our approach to other approaches in the literature in “SLF approach comparison”. Finally, in “Training” the training approach is investigated.

### System overview

Our proposed system is designed to convert a set of features, namely speech derived features, a low resolution image (in case of super-resolution) and other optional features (age, gender and ethnicity) into a high resolution face image. Our system is comprised of two main parts. The first part’s purpose is to create a meaningful feature vector that encapsulates all of the previously mentioned inputs (that are relevant to the output). The second part of the system is designed to act upon the feature vector to generate a high resolution image. Figure 2 shows an overview of the system architecture.

### Feature vector extraction

We employ a fusion-less method to create the required feature vector. We extract several 1-D feature vectors from speech and one hot encode gender and ethnicity. Along with age, we normalize these 1-D feature vectors then stack them to form a two dimensional feature vector of size 256 × 768, which we refer to as “the speech and additional attributes feature vector” (Table 1). As for the low resolution image, it is converted to an 8 × 8 RGB color image and passed to the image generation part of the system.

**Table 1:** The speech and additional attributes feature vector extracted from the feature vector extraction part of the model.

Number of Rows	Extracted from	Scheme	Features
29	Audio	Whole audio	SpeechBrain speaker embeddings
		Sliding window	Pyannote speaker embeddings
		Sliding window	TitaNet speaker embeddings
		Sliding window	SpeakerNet speaker embeddings
190		Sliding window	Librosa Mel spectograms
		Sliding window	Pyaudioanalysis audio features
1		Whole audio	SpeechBrain language class
1	User input	Min-max normalization	Age
1		One hot encoding	Gender
1		One hot encoding	Ethnicity
33	–	–	Zero-padding

This feature vector is a two dimensional matrix of size 256 × 768.

Speech data is processed to extract speaker embeddings, classical audio features and language spoken. Before extraction, all speech clips are either trimmed or replayed to reach a certain length of 24 s.

To extract speaker embeddings we use a multitude of different frameworks (SpeechBrain^32^, Pyannote.audio^33,34^, TitaNet^30^, SpeakerNet^31^). Speaker embeddings are both calculated from the whole length of the audio, as well as, by a sliding window scheme. The resulting speaker embeddings vectors are concatenated along a second dimension resulting in a larger two dimentional feature vector.

To extract classical audio features, we use two frameworks, Librosa^35^ and Pyaudioanalysis^36^. Librosa is used to extract Mel spectrograms in a sliding window fashion, while Pyaudioanalysis is used to extract a multitude of other features (Zero Crossing Rate–Energy–Entropy of Energy–Spectral Centroid–Spectral Spread–Spectral Entropy–Spectral Flux–Spectral Rolloff–MFCCs–Chroma Vector–Chroma Deviation). These features are extracted in a sliding window fashion.

As for age and gender, age is normalized and gender is one-hot encoded. Ethnicity is defined from a set of five possible ethnicities, then is one hot encoded too. The three feature vectors of age, gender and ethnicity are then stacked to form a 2-D additional attributes feature vector.

The 2-D feature vectors resulting from the all of the previously mentioned processes are then stacked and zero-padded to form the “speech and additional attributes feature vector” as shown in Table 1 and whose final size is 256 × 768.

In the case of the super-resolution system, the low resolution image (image guide) is converted to an 8 × 8 RGB image and fed directly to the image generation part of the system as an image feature vector.

### Image generation

The image generation part of the system takes the speech and additional feature vector along with the image feature vector (in case of super-resolution) and generates a high resolution face image (128 × 128). This part is implemented via a diffusion model.

#### Diffusion models

**Fig. 3:**
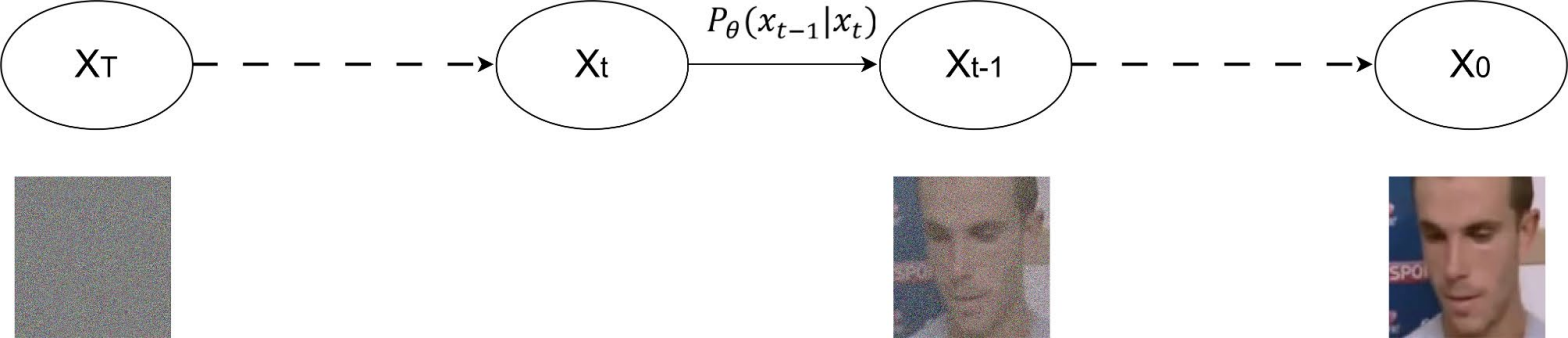
A graphical representation of a diffusion model^11^.

A diffusion probabilistic model is a parameterized Markov chain trained using variational inference to produce samples matching the data after finite time (Fig. 3)^11^. In the context of diffusion models, the conditional scale refers to the adjustment of the diffusion process based on certain conditions or variables. This concept^15,16,37^ is often used to model scenarios where the diffusion rate is not constant but depends on some underlying factors or conditions. The conditional scale can be represented with time as an independent variable (e.g., 0–10) and that scales from$\:\:{P}_{\theta\:}\left({x}_{T}\right|{x}_{t})$, where $\:\theta\:$ represents the model parameters, then the diffusion attribution^37^ will be:

1 $$\:Diffusion\:Attribution=No.\:of\:Samples\:\times\:\:\:Conditional\:Scale$$

Equation (1) yields the influence of data (conditioning) on the diffusion process at any time step $\:t$ including the final instant $\:t=0$. It is worth mentioning that Eq. (1) expresses, in terms of the conditional scale, the entire journey^15,16,37^.

#### Face image generation

Face image generation is achieved using a diffusion model composed of two U-Net models^38^. In the case of face super-resolution, the first U-Net gradually enhances the 8 × 8 RGB color image conditioned by the speech and additional attributes feature vector and produces a 32 × 32 face image of sufficient details. The second U-Net acts upon this produced face image and progressively adds more details conditioned by the same speech and additional attributes feature vector until a face image of resolution 128 × 128 is produced. In the case of a face generation system, a patch of random noise image is presented to the first U-Net instead of the low resolution image.

### SLF algorithm

The steps needed to generate, formally, a high resolution image from the various aforementioned inputs are illustrated in the SLF algorithm given as the following:

# SLF Algorithm.

Input: (speech – low resolution image - age, gender & ethnicity)

Output: (high resolution image)

# Feature Vector Extraction (first part):

- Normalize speech data length to 24 s.
- Extract and stack speaker embeddings using SpeechBrain and pyannote.audio frameworks using whole audio and sliding window approaches, creating a 2-D speaker feature vector.
- Extract and stack classical audio features using Librosa and Pyaudioanalysis, creating a 2-D speech feature vector.
- Normalize age, one-hot encode gender and ethnicity and stack them to form a 2-D additional attributes vector.
- Zero pad the above feature vectors to reach a length of 256.
- Combine the above feature vectors into a final feature vector (256 × 768), formally called the “speech and additional attributes feature vector”, zero-padding as necessary to reach a width of 768.
- Convert the low-resolution image to an 8 × 8 RGB image vector for super-resolution mode only.

# Image Generation (second part):

- If in super-resolution mode, feed the low-resolution image vector and final feature vector into the first U-Net to produce an intermediate 32 × 32 image. Else, start with a random noise image for face generation.
- Process the intermediate image and final feature vector with the second U-Net to generate the high-resolution face image (128 × 128).

### SLF approach comparison

By comparing the SLF approach for speech-conditioned face generation and super-resolution to other similar approaches in the literature, the following differences are apparent:

- The reliance on de-noising diffusion probabilistic models for face image generation instead of GANs and auto-encoders, which were by far the most prevalent for this type of task^17–19^.
- Utilizing standard speaker derived features, namely speaker embeddings and standard classical audio features, which haven’t seen enough use.
- High flexibility design that can work upon different combinations of input in a fusion-less way.
- Architecturally similar approach for both face generation and face super-resolution.
- A modular approach where speech feature extraction methods or face image generation models can be easily replaced.
- Conditional scale value based scalability.

### Training

**Fig. 4:**
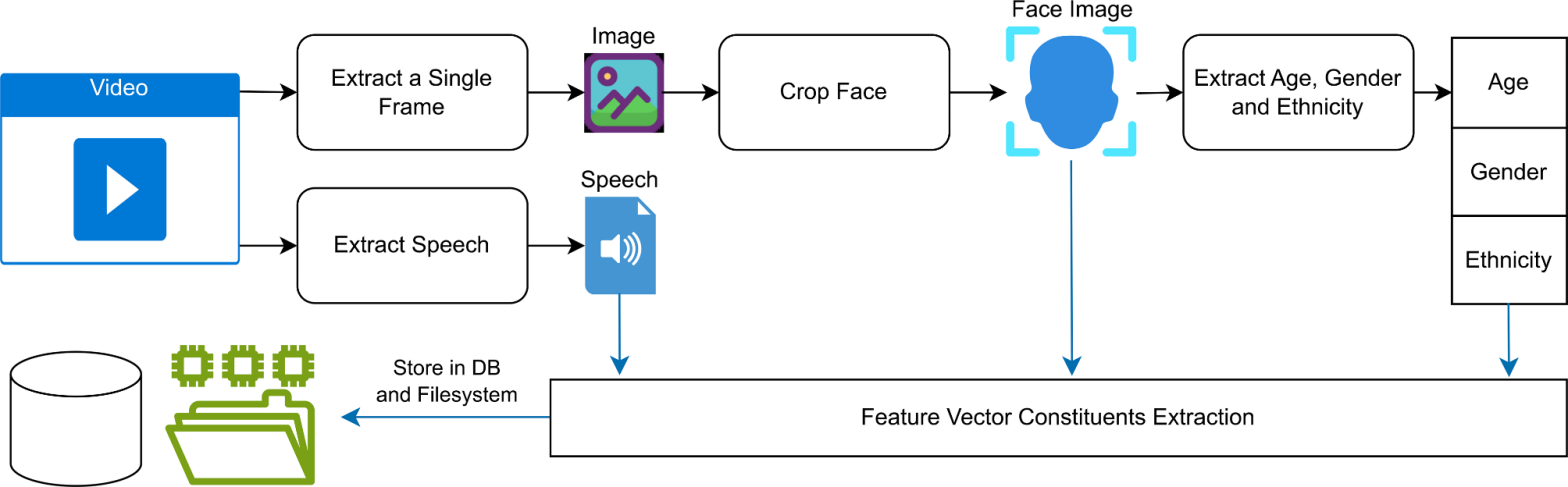
Preprocessing the dataset to extract the feature vector constituents.

**Fig. 5:**
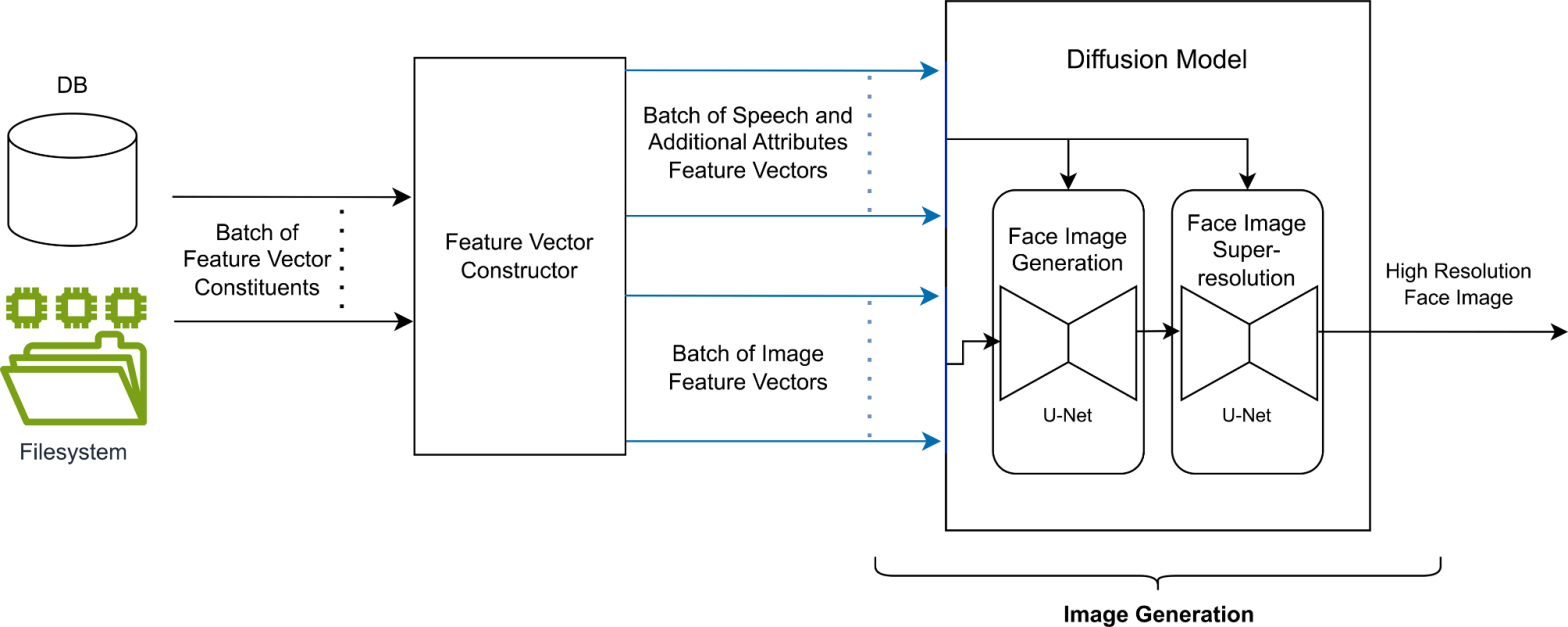
Training the diffusion model using the stored feature vector constituents.

Training the system follows a procedure of selecting a suitable dataset, preprocessing it, pre-computing feature vectors constituents through the feature vector extraction part of the system and storing them, retrieving a batch of feature vector constituents, forming the feature vector and finally training the diffusion model, one U-Net at a time. The procedure is illustrated in Figs. 4 and 5.

The system was trained using the Voxceleb 2 dataset^39^. Voxceleb 2 is a large audio-visual dataset amassed from Youtube. It is composed of about 150 thousand videos of 7.8 s of average length, in which a person of interest is talking. The speakers are formed from a wide range of ethnicities, age-groups, professions and nationalities, and speak different languages with a wide range of accents.

All methods involving the use of the VoxCeleb 2 dataset were carried out in accordance with the relevant guidelines and regulations. The VoxCeleb 2 dataset was collected from publically available Youtube videos and made publically available by its original creators. The Voxceleb 2 dataset is widely used in similar research studies^4,6,8,11^. Its use in this research complies with the appropriate ethical considerations. Experimental protocols involving the use of the VoxCeleb2 dataset in this study did not require additional ethical approval from our institution, as this dataset was collected by the original creators in accordance with the University of Oxford’s Visual Geometry Group (VGG) guidelines and is publically available^40^.

As per the original creators of the VoxCeleb 2 dataset, in compliance with The General Data Protection Regulation (GDPR)^41^, the University of Oxford’s VGG has a data protection exemption under Article 14(5)(b) allowing for the use of this data for scientific research purposes without direct notification to data subjects. This exemption applies when providing such information would involve disproportionate effort, particularly for processing for scientific or historical research purposes. We make use of this exemption for datasets derived from the VoxCeleb 2 dataset.

Speech data and image files were extracted from the various videos, then the images were cropped to include only the face. Age, gender and ethnicity were determined by utilizing the “DeepFace” library^42,43^. Extracted Speech, cropped face images, and age, gender and ethnicity data were then used to generate the constituents of the speech and additional attributes feature vectors, as well as, the image feature vectors as shown in Fig. 4. The accumulated constituents were continually stored in a database and on the filesystem to be ready for training the diffusion model.

Pre-computing the feature vectors this way enables the speedy training of several models without having to compute them again whenever a model needed to be trained. Also, storing the constituents allows selecting only a subset of the constituents to form the final feature vector. This enabled us to train many systems and enabled a thorough ablation study.

When training the diffusion model, a batch of the feature vector constituents are selected then a final feature vector is created and used to train the diffusion model. The diffusion model is trained by first training the first U-Net (image generation) then training the second U-Net (image super-resolution). The procedure is illustrated in Fig. 5.

## Results and discussion

In this section, we first present our set-up and evaluation methodology, next we present our results. After that, a comprehensive ablation study and conditional scale value based scalability are discussed, then we present our results in relation to that of previous works. Finally, we list the limitations of our work and the ethical considerations of this technology.

### Set-up

We utilized the following hardware and software configuration:

Hardware configuration: 12th Gen Intel(R) Core(TM) i9-12900KF, Quadro RTX 6000, VRAM = 24GB, 64 GB RAM.

Sofware configuration: Ubuntu 22.04.2 LTS, Kernal: 5.15.0-25-generic, CUDA 12, Python v3.10.6.

### Evaluation approach

After training a super-resolution SLF system and a face generation SLF system, they are evaluated based on how closely the generated faces produced by the systems resemble their real-face counterparts.

The first step in the evaluation procedure is to decide on a suitable test dataset. This test dataset was selected from the test set split of the VoxCeleb 2 dataset^39^. The VoxCeleb 2 test set contains 118 speakers each in several different videos. To develop our own test dataset, only one video per speaker was selected, cultivating in an evaluation dataset which we refer to as the “SLF Evaluation Dataset”^44^, and which is publically available at https://zenodo.org/records/12706833.

This evalution dataset was then preprocessed in the same manner as the training dataset to produce the feature vector constituents, but unlike during the training procedure, facial embeddings were also extracted from the face image via The Deepface library^42,43^ and stored in a vector database.

Continuing our evaluation procedure, a batch of feature vector constituents are selected from the database and the filesystem, and are then passed to a feature vector constructor which produces a corresponding batch of speech and additional attributes feature vectors and a corresponding batch of image feature vectors. These batches are then passed to the diffusion model in order to generate a corresponding batch of high resolution face images. Thus producing an output that we can finally evaluate.

2 $$\:Recall\:=\:\frac{No.\:of\:correctly\:identified\:persons}{Total\:No.\:of\:persons}$$

The most important evaluation metric of the SLF systems and similar systems is their ability to produce a face image that a facial recognition system can use to link back to the real face image. Thus, the first evaluation metric we include is the recall performance metric, a metric widely used by other research works^3,4,7^. The facial recognition software is also able to return k potential correct persons thus recall, in this case, can be calculated at certain values of k. We decided to calculate recall values at$\:\:k\:=\:\{1,\:3,\:5,\:10\}$. Recall is calculated using Eq. (2).

It is also beneficial to quantify how well certain aspects of the generated face image match those of the real face image, thus gender, ethnicity and age-group recalls are calculated, as well as, the root mean square error of age.

Performance metrics related to image quality are also included. The Average Peak signal to noise ratio (Average PSNR) and the Structural Similarity Index Measure (SSIM) are important for assessing the performance of the super-resolution SLF system and how well it reconstructed the real face image.

### Results

This section contains the results of the evaluation of the SLF system using the metrics discussed in the previous section.

**Fig. 6:**
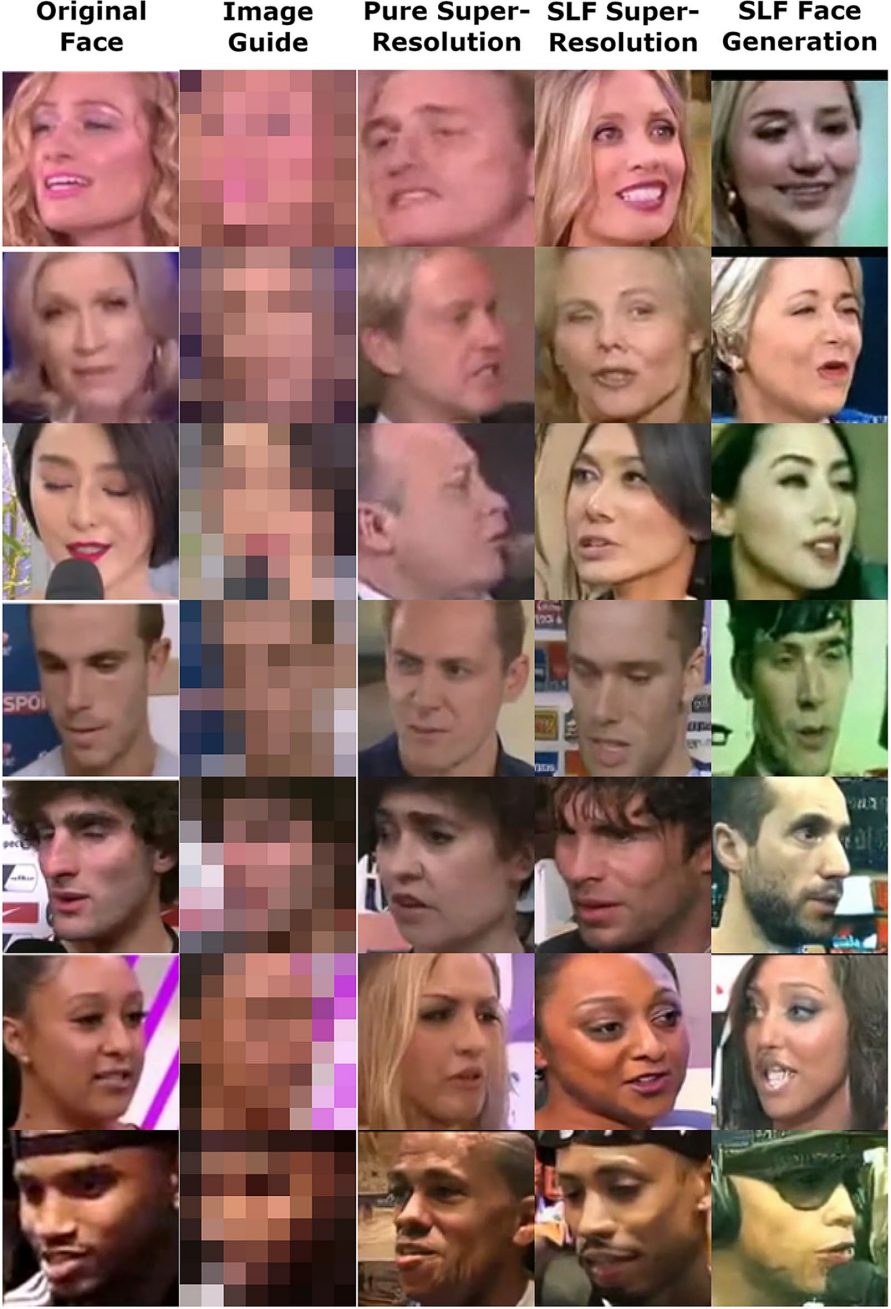
A sample of the output images of a speech conditioned SLF face super-resolution system (column 4) and a speech conditioned SLF face generation system (column 5).

**Table 2:** Recall values for different systems.

System type	Cond. scale	*R*@1 (%)	*R*@3 (%)	*R*@5 (%)	*R*@10 (%)
Random selection	**–**	0.85	2.50	4.20	8.50
Pure super-resolution	**–**	6.80	11.10	16.90	22.90
Super-resolution SLF	8	**16.90**	**30.50**	**33.90**	**45.80**
Super-resolution SLF (2)	4	13.60	20.30	27.10	35.60
Face generation SLF	2	9.30	16.10	23.70	34.70
Face generation SLF (2)	2	7.60	14.40	17.80	28.00

The second entry for the face super-resolution and the face generation SLF systems are calculated without specifying values for the additional attributes, since they are optional inputs.

Significant values are given in bold.

**Table 3:** Gender, ethnicity and age group recalls, as well as, the root mean square errors of age for different systems.

System type	Cond. Scale	Gender recall (%)	Ethnicity recall (%)	Age group recall (%)	RMSE of Age (years)
Pure super-resolution	**-**	78.80	37.30	44.10	7.54
Super-resolution SLF	8	**90.70**	**65.30**	57.60	5.62
Super-resolution SLF (2)	4	85.60	53.40	52.50	5.77
Face generation SLF	2	88.10	58.50	**72.90**	**5.11**
Face generation SLF (2)	2	87.30	51.70	49.20	6.99

The second entry for the face super-resolution and the face generation SLF systems are calculated without specifying values for the additional attributes, since they are optional inputs.

Significant values are given in bold.

**Table 4:** Image fidelity metrics for different systems.

System type	Cond. scale	Average PSNR	Average SSIM
Pure super-resolution	**–**	**16.74**	**0.4**
Super-resolution SLF	8	14.37	0.27
Super-resolution SLF (2)	4	15.71	0.34
Face generation SLF	2	10.46	0.22
Face generation SLF (2)	2	10.57	0.22

The second entry for the face super-resolution and the face generation SLF systems are calculated without specifying values for the additional attributes, since they are optional inputs.

Significant values are given in bold.

Figure 6 shows a sample of the output images of the SLF systems. Performance metrics of the face super-resolution and face generation SLF systems are shown is Tables 2, 3 and 4. Results for random selection algorithms and pure super-resolution (without conditioning) systems are also presented for comparison. The pure super-resolution systems are also constructed in the same way that other SLF systems were built, except that they were conditioned only on the low resolution image.

It is shown that all SLF systems, whether face super-resolution or face generation types, have much better performance than random selection algorithms. All SLF systems also perform better than pure super-resolution systems, except in terms of image fidelity. This is understandable, since conditioning introduces corrupting elements to the final image. However, it is surprising to see that the face generation SLF systems perform better at identifying individuals and their gender, ethnicity and age than pure super-resolution systems.

Face super-resolution SLF systems generally perform better than face generation SLF systems. This improvement is attributed to the extra input, the low-resolution image, which is fed into such systems, especially in terms of image fidelity. Also, providing the gender, ethnicity and age to the SLF systems generally enhances the recall, and gender, ethnicity and age recalls.

Referencing Fig. 6, it is shown that the SLF face super-resolution system generates images which are very similar to the corresponding ground truth images and which follow roughly the same head and face orientation, skin color, skin tone, hair color and hair style. Understandably, The SLF face generation system generates face images that aren’t faithful to the true face images in orientation, hair color, hair style and clothing, but generally generates similar faces to the real faces. The pure super-resolution system’s images demonstrate the importance of audio, and gender, ethnicity and age features to generating faces that resemble true faces.

### Ablation study

Several other different systems have been trained in order to study the effect of the various inputs (speaker embeddings–classical audio features–language spoken–low resolution image–ethnicity–gender–age) on the intended output. To ease referring to the different systems, from here on we refer to the SLF face super-resolution model as the “Full Input” model, since it is trained using all the previously mentioned inputs. All other ablation systems are named in relation to the full input model.

**Table 5:** Recall values for the different ablation systems.

#	System	Cond. scale	*R*@1 (%)	*R*@3 (%)	*R*@5 (%)	*R*@10 (%)
–	Random selection	**–**	0.85	2.50	4.20	8.50
1	Only image guide	**–**	6.80	11.10	16.90	22.90
2	Full input	8	**16.90**	**30.50**	**33.90**	**45.80**
3	No image guide	2	9.30	16.10	23.70	34.70
4	No speaker embedding	8	9.30	13.60	16.10	24.60
5	No audio	4	9.30	15.25	25.40	31.40
6	Only audio	2	7.60	14.40	17.80	28.00
7	Speaker embedding only	4	8.50	13.60	17.80	29.60
8	No additional attributes	4	13.60	20.30	27.10	35.60

Significant values are given in bold.

In order to easily quantify the effects of the various inputs on the performance of the system and to summarize that effect into a single metric, we measure a weighted average of the recall increase percentages. This is done by first measuring the recall increase by percentage when a particular input is added to the list of inputs to the system. This recall increase is different for every value of $\:k$ (Table 5), and rather than calculating the average of these recall increase percentages, we calculate a weighted average. This is a better indication of the influence of the added input, since performance at smaller values of $\:k$ is more important than at larger values of $\:k$.

3 $$\:{R}_{inc,\:k=c}^{Due\:to\:input\:P}=\:\frac{{R}_{k=c}^{all\:features}-\:{R}_{k=c}^{without\:input\:P}}{{R}_{k=c}^{without\:input\:P}}\:\times\:100\text{\%}$$

4 $$\:S=\frac{{10R}_{inc,k=1}+{5R}_{inc,k=3}+{3R}_{inc,k=5}+{R}_{inc,k=10}}{10+5+3+\:1}$$

Equation (3) illustrates how to calculate the recall increase by percentage due to input$\:\:P$, abbreviated by $\:{R}_{inc,k=c}^{Due\:to\:input\:P}$, from the recall values$\:\:{R}_{k=c}^{all\:features}$ and$\:{\:R}_{k=c}^{without\:input\:P}$. Equation (4) illustrates how to calculate the weighted average of the recall increase percentages, abbreviated by$\:\:S$ (significance). We conclude that the effect of the input on the performance of the system is negligible when $\:S$ is zero, minor when$\:\:0<S\le\:20\%$, moderate when$\:\:20\%<S\le\:40\%$, considerable when$\:\:40\%<S\le\:60\%$, significant when $\:60\%<S\le\:80\%$ and profound when$\:\:80\%<S$.

#### The effect of the low resolution image

The effect of the low-resolution image is significant ($\:S=75\%$); Comparing system No. 2 and system No. 3, the recall performance of the “Full Input” system was much greater than that of the “No Image Guide” system. Ethnicity, gender, and age recall values were also slightly higher.

Understandably, image fidelity metrics greatly suffered without the image guide; the diffusion model is unable to accurately reconstruct elements such as hair, hairstyle, clothes, accessories, facial hair, and background color (Table 6, 7).

**Table 6:** Gender, ethnicity and age recall values for the different ablation systems.

#	System	Cond. scale	Gender recall (%)	Ethnicity recall (%)	Age group recall (%)	RMSE of age (years)
1	Only image guide	–	78.80	37.30	44.10	7.54
2	Full input	8	**90.70**	65.30	57.60	5.62
3	No image guide	2	88.10	58.50	**72.90**	**5.11**
4	No speaker embedding	8	74.60	77.10	51.70	5.8
5	No audio	4	73.70	**78.80**	55.10	5.21
6	Only audio	2	87.30	51.70	49.20	6.99
7	Speaker embedding only	4	89.00	47.50	41.50	7.54
8	No additional attributes	4	85.60	53.40	52.50	5.77

Significant values are given in bold.

**Table 7:** Image fidelity metrics for the different ablation systems.

#	System	Cond. scale	Average PSNR	Average SSIM
1	Only image guide	–	**16.74**	**0.4**
2	Full input	8	14.37	0.27
3	No image guide	2	10.46	0.22
4	No speaker embedding	8	16.29	0.37
5	No audio	4	16.5	0.39
6	Only audio	2	10.57	0.22
7	Speaker embedding only	4	10.52	0.2
8	No additional attributes	4	15.71	0.34

Significant values are given in bold.

#### The effect of audio

The effect of audio is significant ($\:S=77\%$). By comparing the performance of system No. 2 (“Full Input”) to system No.5 (“No Audio”), it is shown that the “No Audio” system had significantly lower recall values in comparison to the full input system. Gender, ethnicity and age accuracies were influenced in differing ways, but are generally better in the “Full Input” system. Image fidelity metrics were better in the “No Audio” system owing to the corrupting influence audio has on the output images in the “Full Input” system.

It is also beneficial to compare the performance of the “No Image Guide” system (No. 3) to the “No Speaker Embeddings” system (No.4) and to the “No Audio” system (No. 5). It is shown that system performance decreased more when speaker embeddings were removed from the input list compared to when audio features or image guides were removed. This illustrates that speaker embeddings have a greater effect than low-resolution images (8 × 8), while also highlighting the importance of both for the task of person recognition.

#### The effect of speaker embeddings

The effect of speaker embeddings is profound ($\:S=98\%$) and they are sufficient audio features for our task. By Comparing the performance of the “Full Input” system (No. 2) to the performance of the “No Speaker Embeddings” system (No. 4), it is shown that the recall values greatly increase when training is done while including the person’s speaker embeddings as an input. Gender, ethnicity and age recall values also increase.

Image fidelity metrics are better in the “No Speaker Embeddings” system. This can be attributed to the higher influence of the speaker embeddings on the output image while having no information on other elements of the image such as clothing.

It is clear that although the two systems are trained with audio as an input, speaker embeddings easily encapsulate the identity of the person, allowing the diffusion model to better represent the person’s face at the cost of image fidelity metrics.

Comparing the weighted average of recall increase percentages due to audio ($\:S=77\%$) to that due to speaker embeddings ($\:S=98\%$). It is also evident that the diffusion model struggles to extract relevant information from the classical audio features and language features pertaining to our task. This could be due to the model requiring much more training or the possibility that the chosen classical audio features, their parameters, and schemas were unsuitable. Different classical audio features or alternative parameters and schemas may need to be considered.

#### The effect of the values of gender, ethnicity and age

The effects of including gender, ethnicity and age in the training and testing data are moderate ($\:S=31\%$). This can be deduced by comparing the performance of system No. 8 (“No Additional Attributes” system) to system No.2 (“Full Input” system).

### Conditional scale value-based scalability

**Table 8:** The performance of different systems with respect to different conditional scale values.

System	Cond. scale	*R*@1 (%)	*R*@3 (%)	*R*@5 (%)	*R*@10 (%)	Gender accuracy (%)	Ethnicity accuracy (%)	Age group accuracy (%)	RMSE of age (years)	Average PSNR	Average SSIM
Random selection	–	0.85	2.50	4.20	8.50	–	–	–	–	–	–
Only image guide	–	6.80	11.10	16.90	22.90	–	–	–	–	–	–
Full input	0	5.10	7.60	14.40	20.30	71.20	46.60	42.40	7.55	**16.72**	**0.4**
	2	9.30	18.60	28.80	42.40	89.00	62.70	55.10	5.87	16.52	0.38
	4	7.60	22.00	28.00	39.00	91.50	64.40	**58.50**	**5.05**	15.57	0.34
	6	**16.90**	24.60	31.40	**46.60**	87.30	64.40	**58.50**	5.59	14.96	0.3
	8	**16.90**	**30.50**	**33.90**	45.80	**90.70**	**65.30**	57.60	5.62	14.37	0.27
Speaker embedding only	0	1.70	3.40	4.20	10.20	60.20	45.80	47.50	8.52	10.95	**0.24**
	2	**8.50**	12.70	17.80	29.70	**89.00**	46.60	43.20	**6.93**	**11.11**	0.23
	4	**8.50**	13.60	17.80	29.60	**89.00**	47.50	41.50	7.54	10.52	0.2
	6	6.80	**15.30**	**21.20**	31.40	85.60	54.20	**48.30**	8.31	10.38	0.18
	8	4.30	8.50	14.50	**32.50**	88.00	**47.90**	43.60	7.96	10.26	0.16

Significant values are given in bold.

**Fig. 7:**
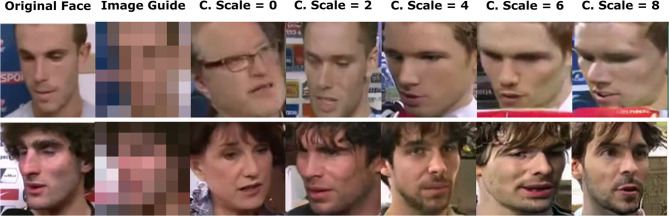
Output images from a “full input” system at different conditional scales.

The conditional scale value is used as a cheap alternative to the costly process of scaling the model by increasing the number of neural network weights then retraining it. The conditional scale value dictates how strongly the conditional values influence the patch of noisy image or the low resolution image used as a basis for the generation of the output image. Zero value of the conditional scale instructs the diffusion model to completely ignore the conditional values. Unnecessary high values introduce inaccurate changes to the output image and corrupts it.

Referencing Table 8, it is shown that recall values, age, ethnicity and age recall values, and image fidelity metrics change rapidly when changing the conditional scale value. The best performance can be achieved at a certain conditional scale value. However, this value is determined experimentally. At zero conditional scale value all models, which were trained with the low resolution images, behave like a pure super-resolution model, and all models, which weren’t trained with the low resolution images, behave as a random selection model.

It is shown that when increasing the conditional scale value, image fidelity tend to deteriorate. That happens due to higher degree of influence the conditional values exert on the output images, which sometimes change the output image in an inaccurate way. Output images from a “Full Input” system at different conditional scales are shown in Fig. 7.

**Fig. 8:**
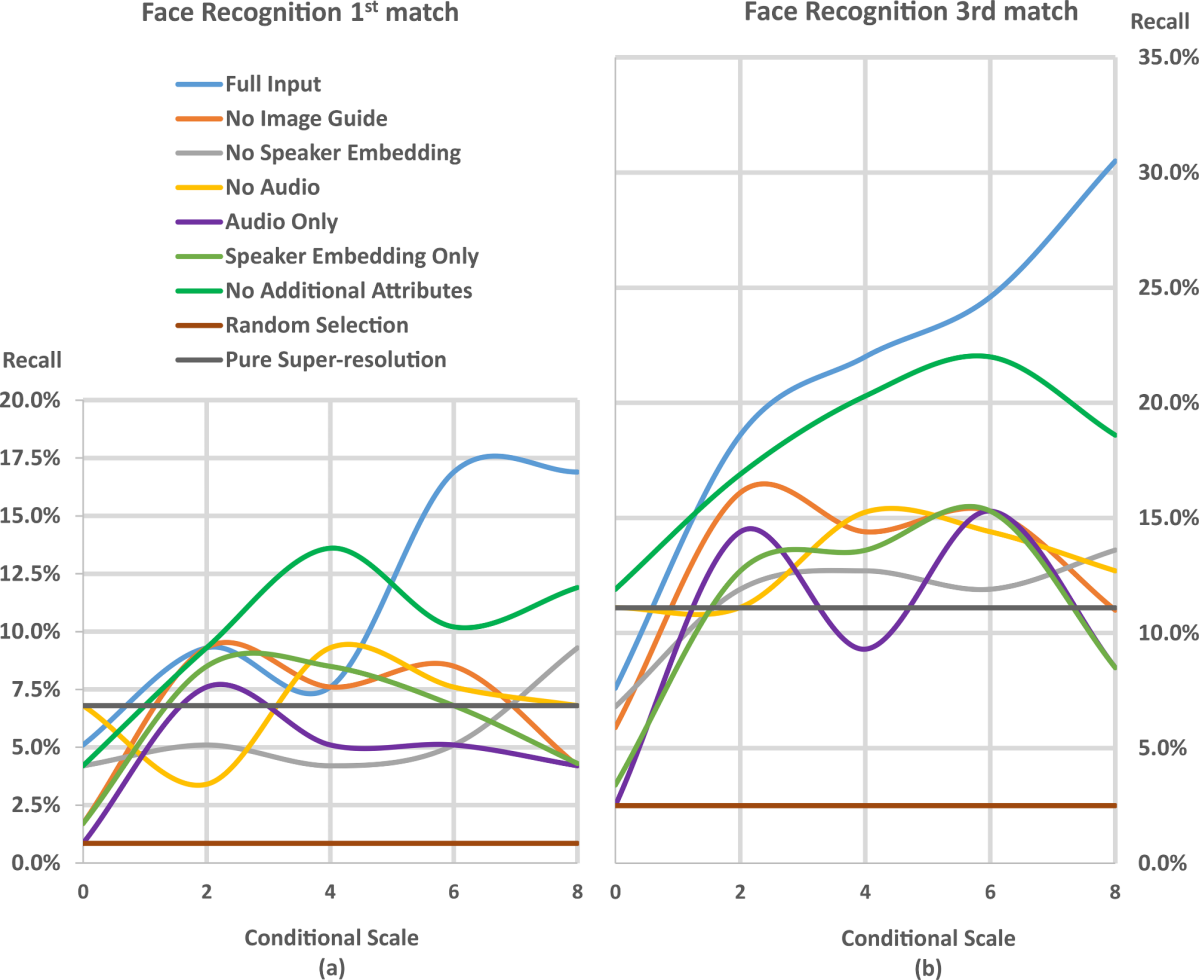
Recall values at 1 potential candidates (**a**) and 3 potential candidates (**b**) at different conditional scale values and different ablation SLF systems.

As we performed extensive experimentations on the various SLF systems, altering only the conditional scale value which influences the diffusion process, we obtained recall values at 1 potential candidate (Fig. 8a) and 3 potential candidates (Fig. 8b). Despite the numerous findings, we report only the essentials of those pertaining to the face recognition performance at 1 potential candidates.

- The “Full Input” system is the best performing system at conditional scale values from 1 to 2 and 5 to 8.
- By comparing the recall values of the “Full Input” system to those of the “No Image Guide” system, the performance deteriorates. This is due to the absence of the low resolution image in the input.
- Likewise, the performance of the “No Audio” system deteriorates due to the absence of speech data.
- The “No Additional Attributes” system is the best performing system at conditional scale values from 2 to 5.
- The “Audio Only” system performs worse than the “Speaker Embeddings Only” system at all conditional scale values.
- The “Speaker Embeddings only” system is the only uni-feature system presented. Its performance increases in a stable fashion from conditional scale values 0 to 3, then decreases in a stable fashion from conditional scale value 3 to 8. Its performance curve as shown in Fig. 8.a is different from the performance curves of all the other systems in this regard.
- As expected, all SLF systems perform better than a random selection curve.
- All systems that have recall values higher than that of the pure super-resolution system at certain conditional scale values achieve these higher recall values due to the information contained within the multi-modality. However, when the recall values are lower, this can be attributed to one of two reasons; either the information corrupts the output image beyond usefulness (relatively high conditional scale values), or the information insufficiently affects the output image (relatively low conditional scale values or too few inputs that can’t compensate the exclusion of the image guide, as in the case of the “Audio Only” system).

### Position of our systems relative to other systems

Surveying the literature shows that there is a great distinction in the types of models used for speech2face systems. Some systems accept audio only^3^, while others primarily accept a low resolution face image along with an audio of the person^7^. The audio length accepted also differs from one system to another^3–10^. As for the output image, the color mode (grayscale vs. colored), output image resolution (64 × 64, 128 × 128 or others) and whether the face image is normalized or not, differs from one system to another.

**Table 9:** Performance of different speech conditioned face super-resolution systems.

Model	Inputs	Size of evaluation dataset	Recall @1 (%)	Gender accuracy (%)	RMSE of age (years)	PSNR
Learning to have an ear for face super-resolution^7^	Audio and 8 × 8 low resolution image		15.67	93.11	3.68	19.97
SLF face super-resolution	Random	Unseen 118 speakers, 1 video per speaker	0.85			
	Audio, 8 × 8 low resolution image and additional attributes		16.90	90.70	5.62	14.37
	Audio and 8 × 8 low resolution image		13.60	85.60	5.77	15.71

The output image has a resolution of 128 × 128 pixels.

**Table 10:** Performance of different speech conditioned face generation systems.

System	Inputs	Size of evaluation dataset	Output image	Recall @1 (%)	Recall @3 (%)	Recall @5 (%)	Recall @10 (%)
Speech2Face: learning the face behind a voice^3^	Random	5000		1		5	10
	Audio 3 s		Normalized	8.54		24.8	38.54
	Audio 6 s			10.92		30.6	45.82
Speech fusion to face: bridging the gap between human’s vocal characteristics and facial imaging^4^		378	64 × 64	5.80		20.40	36.70
			128 × 128	5.00		19.40	32.30
			64 × 64 and 128 × 128	6.10		18.80	35.40
Speaker embedding SLF face generation	Random	118	128 × 128 non-normalized	0.85	2.50	4.20	8.50
	Audio and additional attributes			9.30	16.10	23.70	34.70
	Audio			8.50	13.60	17.80	29.60

The previously mentioned factors do not represent all the differences; other differences can include the type, size and diversity of the dataset used for training, and more importantly, the type, size and diversity of the evaluation dataset. It is also crucial to consider whether the evaluation dataset contains the same individuals as the training dataset. All these factors can vary greatly from one system to another in the literature.

Another difference lies in whether a certain person appears multiple times in the evaluation dataset. Reporting random selection recall accuracies give a sense of how many times a person appears in a dataset.

Additionally, The performance metrics used to evaluate the different models differ greatly, and some performance metrics, for example: gender, ethnicity and age accuracy, rely on the accuracy of other pre-trained models. Each paper uses a different combination of these models. The performance of that combination must surely affect the performance metrics.

For all the above reasons, it is generally difficult to accurately compare the different systems. Tables 9 and 10 attempt to present the relative position of the SLF systems rather than being a formal unbiased objective comparison.

### Limitations

As previously mentioned, our ultimate goal is to enhance person recognition recall through either face generation or face super-resolution, but it is imperative that we explore the limitations of these technologies.

Tables 9 and 10 show that using speech-conditioned face super-resolution and generation systems alone to identify a single individual is unreliable. However, recall values improve as the number of potential individuals increases, offering a useful compromise for person recognition tasks.

Disadvantages to our approach include the costly and slow training process of the diffusion models^14^, as well as, needing large and fast storage media to store the large amounts of videos and preprocessed data.

Our approach relies on several different models in a modular fashion. While this architecture makes our systems extremely flexible, giving the capability to replace some of these models with ease, as well as, enabling us to produce an extensive ablation study, it introduces complexity that other end to end solutions do not present.

Additionally, our approach relies on conditional scale value based scalability which offers a great alternative to traditional parameter amount based scalability that is costly and difficult to use. But it is noted that traditional scalability techniques have their uses and can be used in conjunction with conditional scale value scalability.

Finally, it is worth mentioning that recently multimodal large language models (MLLM) are booming^45^. They can efficiently and creatively perform multimodal tasks. The astonishing capabilities of MLLM can also be used for multimodal speech conditioned face super-resolution and face generation.

### Ethical considerations

It is imperative that we explore the ethical considerations that result from the misuse of our approach and similar approaches, a topic we reiterate along with other research works^3,4^. This misuse can result from wrongly interpreting the results or from using them for malicious purposes.

One such potential for misuse is the reliance on derived image aspects that cannot be reliably derived from the inputs fed to these systems, such as, deriving hair color, hair style, etc. (Fig. 6), and then utilizing or building upon these image aspects.

Privacy concerns related to our proposed approach and other similar approaches mirror those of face recognition and speaker recognition systems. Several research works were produced that try to list, analyze and solve the complex privacy concerns related to facial recognition technology^46,47^. Some of these concerns that resonate with speech conditioned face generation and face super resolution technology include:

- Maintaining transparency of how this technology works and the data by which it was trained.
- The ability to challenge outcomes obtained from such systems.
- Protecting users’ data, maintaining its confidentiality, and upholding consent and ownership.
- Making sure that such technology is governed by local laws and policies that simultaneously permit and limit its use to well-intended legal applications, as well as, ban and actively stop its use in illegal activities.
- Regulating bodies are needed to make sure that the preceding points are met.

The issue of bias is highly present in speech conditioned face generation and super-resolution systems. This is due to the generative and correlative nature of such systems, and due to the fact that such systems generate some images aspects that are unrelated to the input data but relate highly to the training data (e.g., clothes). It is imperative that special attention is paid when constructing a training set and when evaluating system performance.

Finally, speech conditioned face generation and super-resolution technology have the potential to allow malicious actors to infringe upon user’s privacy by revealing a person’s face, identity and other characteristics without the user’s consent. This issue is further exacerbated if the face image were to be used for unauthorized access, deepfake technology^48^ or other such misuse. Of course, it is noted that the limitations of speech conditioned face generation and super resolution technology restrict these uses greatly.

It is worth noting that all face images in this study either originate from the VoxCeleb 2 dataset^39^ or from the outputs of SLF systems that were trained on the VoxCeleb 2 dataset, and using inputs that originate from the VoxCeleb 2 dataset.

## Conclusion

This paper has presented the SLF system as a multimodal, modular speech-conditioned face image generation and super-resolution system capable of handling various unimodal case studies or a combination of them. The SLF system is comprised of two main components: a feature vector extraction component, which is fusion-less and flexible, and an image generation component, which exploits U-Nets to build a conditional diffusion model in order to generate the required face. Moreover, every input’s influence on the SLF system’s performance was measured experimentally, as was the effect of the diffusion’s model conditional scale value parameter, so that the SLF can achieve consistent scalability. By making use of SLF we found out the following:

1. The effects of the audio signals on the output face image are profound and are more pronounced than those of the low resolution images (8 × 8), whose effects are still significant. Moreover, Speaker embeddings can be relied upon as sufficient audio features for our task. However, the effect of gender, ethnicity and age inputs are moderate.
2. Conditional scale values greatly affect the SLF system’s behavior and performance. Actually, it has been found out that for each system (acting on different combination of inputs) the conditional scale values affect the diffusion performance differently.

## Data Availability

The code used to build, train and evaluate the SLF systems is publically available on GitHub at https://github.com/AhmedGamal411/DiffusionSpeech2Face^49^ and is licensed under the Creative Commons Attribution 4.0 International License.The data that support the findings of this study are derived from the VoxCeleb 2 dataset, which is publicly available and licensed under the Creative Commons Attribution-ShareAlike 4.0 International License. The original VoxCeleb 2 dataset can be accessed at https://www.robots.ox.ac.uk/~vgg/data/voxceleb/vox2.html.In this study, we used the test set split of the VoxCeleb 2 dataset to construct a new dataset, referred to as the “SLF Evaluation Dataset”. The SLF Evaluation Dataset is publicly available and is distributed under the same Creative Commons Attribution-ShareAlike 4.0 International License as the original VoxCeleb 2 dataset. This dataset can be accessed at https://zenodo.org/records/12706833. Researchers are free to use, redistribute, and build upon this modified dataset, provided appropriate credit is given, and any derivative works are distributed under the same license.
